# Integrating *In silico* perturbation with multilayer omics to decode regulatory networks in cancer immunity: a new frontier in precision oncology

**DOI:** 10.3389/fimmu.2026.1857447

**Published:** 2026-06-05

**Authors:** Haifang Chen, Zhiyu Chen, Mingna Sun, Lu Liang

**Affiliations:** 1 Operations Management Department, The Affiliated Traditional Chinese Medicine Hospital, Guangzhou Medical University, Guangzhou, China; 2 The Fifth Affiliated Hospital, Key Laboratory of Molecular Target & Clinical Pharmacology, National Medical Products Administration (NMPA), and the State Key Laboratory of Respiratory Disease, The School of Pharmaceutical Sciences, Guangzhou Medical University, Guangzhou, China

**Keywords:** foundation models, in silico knockout, multi-omics integration, tumor immunology, tumor microenvironment

## Abstract

The traditional paradigm of linking single genes to individual phenotypes is being replaced by a systems-level framework to understand the complexity of the tumor microenvironment. In this context, in silico knockout has emerged as a powerful computational approach to predict system-wide responses to genetic or cellular perturbations. This review summarizes how multilayer regulatory information across the genome, transcriptome, proteome, and metabolome can be integrated into computational models for virtual perturbation analysis. We outline major multi-omics data sources, including bulk, single-cell, and spatial omics, and emphasize how these data are transformed into model-compatible inputs such as constraint-based matrices and latent embeddings. We then discuss the evolution of in silico knockout methodologies, from genome-scale metabolic models and flux balance analysis to advanced deep learning frameworks that enable the prediction of non-linear and unseen perturbations. The integration of spatial transcriptomics further extends these approaches to tissue-level modeling of cell-cell interactions. In tumor immunology, these methods facilitate the identification of immune regulatory genes, the analysis of immune evasion mechanisms, and the prioritization of therapeutic targets. Despite current challenges in multi-omics integration and biological complexity, in silico knockout provides a promising framework for advancing precision immunotherapy.

## 1 Introduction

The emergence of cancer immunotherapy has fundamentally transfigured the therapeutic paradigm for malignant tumors. Its primary objective is to reinvigorate the host’s immune system, enabling it to recognize and eliminate malignant cells. Clinical breakthroughs in immune checkpoint blockade (ICB) and adoptive cell therapies, such as CAR-T and CAR-NK, have offered durable clinical benefits for patients with various previously intractable late-stage hematological and solid tumors (1, 2). However, a significant proportion of patients still exhibit primary or acquired resistance, a phenomenon largely attributed to sophisticated mechanisms of immune evasion. Within the tumor microenvironment (TME), malignant cells do not exist in isolation but orchestrate a suppressive ecosystem. This immune-shield is maintained by specialized cell populations, such as immunosuppressive *SPP1+* or *TREM2+* macrophages and hypoxia-induced fibroblasts, which collectively hinder the infiltration and effector function of cytotoxic lymphocytes (3, 4).

The resilience and adaptability of this ecosystem are governed by complex, multilayered regulatory networks that bridge genetic, epigenetic, and metabolic dimensions. At the epigenetic level, specific modifications such as histone lactylation (H3K18la) have been identified as pivotal spatiotemporal regulators that link glycolytic flux to the transcriptional activation of oncogenes and immune-evasive signatures (5). Concurrently, metabolic reprogramming serves as a fundamental pillar of immune modulation; for instance, the availability of cytosolic acetyl-coenzyme A acts as a rheostat for mitophagy, while mannose metabolism can drastically reshape T cell differentiation and anti-tumor potency (6, 7). Furthermore, the spatial architecture of the TME, as exemplified by paracrine signaling loops such as the hypoxia-induced Wnt5a secretion from fibroblasts, reinforces these regulatory circuits, creating a self-sustaining niche that promotes cancer progression (8).

Despite the power of functional genomics, particularly genome-wide CRISPR screens, conventional experimental approaches face substantial bottlenecks in deconstructing these multidimensional networks. Physical knockout screens in primary human immune cells are not only resource-intensive and technically demanding but also constrained by the curse of dimensionality, where the astronomical number of gene combinations and their context-specific effects within a dynamic TME cannot be experimentally tested (9, 10). Furthermore, traditional assays often provide only a static snapshot, failing to capture the metabolic flux or the transient regulatory states of cells during the progression of treatment.

**Figure 1:**
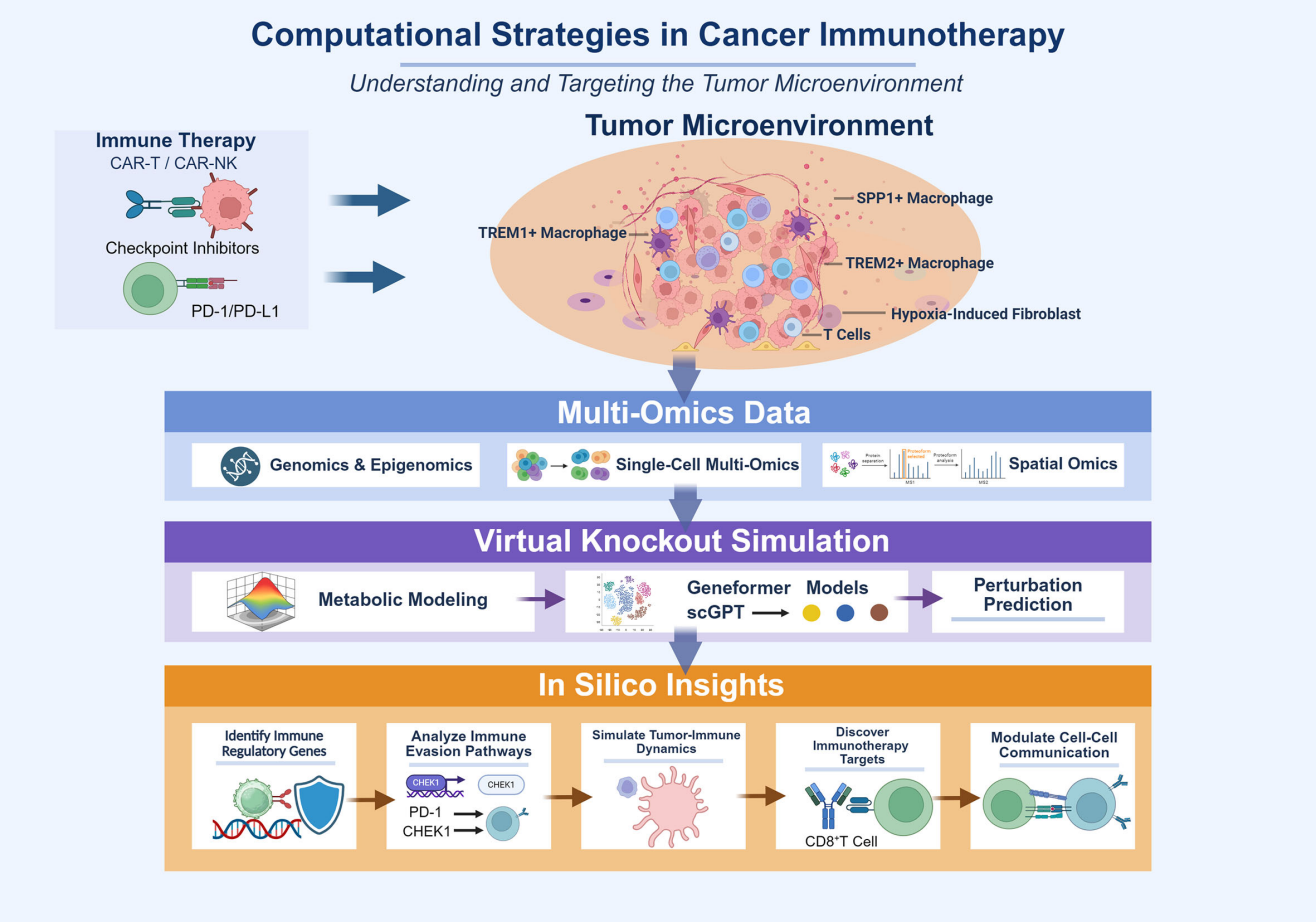
Integrated computational workflow of in silico knockout in tumor immunology.

To bridge this gap, computational modeling and in silico perturbation (virtual knockout) have emerged as indispensable frontiers in precision oncology. By leveraging large-scale foundation models (e.g., Geneformer) pre-trained on vast single-cell datasets, or utilizing Genome-Scale Metabolic Models (GEMs), researchers can now simulate the systematic deletion of genes within a digitized cellular framework (11, 12). These virtual perturbations allow for the high-throughput identification of master regulators that can revert drug-resistant states or sensitize tumors to immunotherapy by predicting the downstream impact on transcriptional meta programs and metabolic homeostasis. This review aims to synthesize recent advancements in integrating multilayered omics with in silico strategies, highlighting how these digital experiments are redefining our ability to decode and therapeutically target the regulatory essence of cancer immunity. The integrated workflow of in silico knockout, spanning from multi-omics data acquisition to virtual perturbation and clinical validation, is illustrated in **Figure 1**.

## 2 Multilayer regulatory networks and multi-omics integration

The core of systems biology lies in the shift from a reductionist view of single genes mapping to single phenotypes to a holistic understanding of how biological entities interact across multiple scales. In the context of cancer immunity, decoding the landscape requires a systematic integration of information flowing through the central dogma and beyond, encompassing the genome, transcriptome, proteome, and metabolome. The comprehensive architecture of these regulatory layers, along with their respective multi-omics data sources and computational integration strategies, is systematically summarized in **Table 1**.

**Table 1:** Multilayer regulatory architecture, data sources, and computational integration strategies.

Layer / component	Biological features	Data type & source	Integration strategy	Output for downstream models	References
Genomic & Epigenomic Layer	DNA mutations, structural variations, chromatin accessibility (e.g., lactylation)	Bulk sequencing (TCGA, GEO), epigenomics	Feature extraction, mutation profiling	Gene-level constraints, regulatory priors	(5)
Transcriptional Layer	Gene expression programs (e.g., EMT-inflammatory, Prolif-stress)	Bulk RNA-seq, scRNA-seq	Dimensionality reduction, clustering	Expression matrices, cell states, embeddings	(3)
Proteomic & Post-translational Layer	Protein abundance, ubiquitination (e.g., Cop1), signaling modulation	Proteomics, inferred from transcriptomics	Network inference, activity scoring	Signaling activity scores, regulatory networks	(4)
Metabolic Layer	Metabolites (e.g., acetyl-CoA, mannose), metabolic flux	Metabolomics, inferred flux models	Constraint-based modeling (GEMs)	Stoichiometric matrices, flux distributions	(6, 7)
Bulk Omics Data	Population-level genomic & clinical patterns	TCGA, GEO	Statistical modeling, biomarker discovery	Gene signatures, risk models	(13, 14)
Single-cell Omics	Cellular heterogeneity (e.g., SPP1^+^ macrophages)	scRNA-seq, scATAC-seq	Clustering, trajectory inference	Cell-type-specific features	(15, 16)
Spatial Omics	Cell-cell proximity, niche interactions (e.g., Wnt5a signaling)	Spatial transcriptomics	Spatial mapping, neighborhood analysis	Spatial graphs, interaction networks	(17–19)
Network Modeling (GEMs)	Gene–metabolism linkage	Multi-omics integrated	Constraint-based reconstruction	Flux balance models (FBA-ready)	(2)
Machine Learning Models	Non-linear feature extraction	Multi-omics datasets	Representation learning	Latent embeddings	(13)
Deep Learning / Transformers	Gene regulation grammar, perturbation prediction	Large-scale omics data	Pretraining + fine-tuning	Predictive models for knockout simulation	(13, 14)
Bridging to In Silico Knockout	Conversion to model-ready inputs	Integrated multi-omics	Encoding into matrices / embeddings	Inputs for GEMs & AI perturbation models	(2, 13, 14)

### 2.1 The architecture of multilayer networks

Biological regulation is inherently hierarchical and interconnected. A multilayer network represents these interactions as a series of coupled layers. At the foundation lies the genomic and epigenomic layer, where structural variations and chromatin modifications, such as H3K18la lactylation, dictate the accessibility of genetic information and set the stage for subsequent regulation (5, 13). This is followed by the transcriptional layer, the dynamic output of the genome where transcription factors coordinate the expression of meta programs defining cell identity, such as the *EMT-inflammatory* or *Prolif-stress* states observed in malignant tissues (3, 14). The functional execution then occurs at the proteomic and post-translational layer, where proteins undergo modifications such as ubiquitination by E3 ligases like Cop1 in order to modulate signaling stability and immune cell infiltration (4, 20). Finally, the metabolic layer represents the ultimate functional state where small molecules like acetyl-coenzyme A or mannose act as both fuel and signaling metabolites, providing real-time feedback to the epigenetic and transcriptional layers (6, 7, 21).

### 2.2 Data sources for integrative analysis

The construction of these high-fidelity networks relies on high-dimensional data harvested from diverse repositories. Bulk omics data from sources like The Cancer Genome Atlas (TCGA) and GEO provide a pan-cancer overview of genomic alterations and clinical outcomes, which is essential for identifying broad immunosuppressive drivers like CHEK1 (22, 23). To resolve the inherent heterogeneity of TME, single-cell omics (e.g., scRNA-seq and scATAC-seq) are utilized to identify specific rare cell populations, such as *SPP1+* macrophages, that drive immune evasion (15, 24). Furthermore, the addition of spatial transcriptomics provides a crucial spatial coordinate that reveals how the proximity of various cell types, such as hypoxia-induced fibroblasts, triggers signaling loops like the Wnt5a axis that promote cancer progression (17).

### 2.3 Methodologies for multi-omics integration

Translating these disparate data types into a unified regulatory map requires advanced computational frameworks. Network modeling techniques, particularly GEMs, utilize biochemical constraints to link genomic data with metabolic flux, enabling the simulation of how gene knockouts perturb the entire system’s equilibrium (25). In parallel, machine learning and deep learning are increasingly employed to extract non-linear features from multi-omics data. Interpretable AI models, such as PRIME, and transformer-based architectures like Geneformer allow researchers to prioritize mutational intolerance or predict treatment responses by learning the latent grammar of gene regulation across different biological layers (26–28).

Crucially, these integration methodologies serve as the functional bridge to perturbation analysis. By converting raw multi-omics datasets into structured inputs, including metabolite-reaction stoichiometric matrices for mechanistic models and high-dimensional latent embeddings for deep learning architectures, these frameworks provide the necessary starting configurations for the virtual knockout simulations described in the following section.

## 3 In silico knockout: from metabolic flux to AI-driven perturbation simulation

The field of *in silico* knockout has evolved from simple logic-based gate simulations to a sophisticated multi-dimensional framework capable of predicting complex non-linear responses within the tumor ecosystem. By leveraging computational models to simulate the absence of genes or cellular components, researchers can perform high-throughput screening and identify therapeutic vulnerabilities without the immediate need for exhaustive wet-lab experimentation.

The efficacy of these simulations depends on the precision of the integrated data inputs derived from the aforementioned omics layers, which define the baseline state of the digital twin prior to perturbation.

### 3.1 Model-based virtual knockout via GEMs

GEMs represent a cornerstone in understanding the metabolic reprogramming that drives cancer progression and drug resistance. This approach utilizes integrated transcriptomic and proteomic profiles as regulatory constraints to tailor generic metabolic templates into patient-specific models. In the context of breast cancer research, Flux Balance Analysis (FBA) serves as the primary engine for model-based virtual knockouts. FBA translates biochemical reactions into mathematical constraints, allowing for the calculation of steady-state flux distributions across the entire metabolic network. Recent frameworks have integrated single-gene knockout simulations with clustering algorithms like UMAP and k-means to identify metabolic targets that can revert the flux state of drug-resistant cells back to a drug-sensitive parental phenotype (29). Utilizing tools such as the COBRA toolbox or RAVEN, researchers can integrate patient-specific transcriptomic data to build personalized models. The hallmark of this approach is its ability to predict the impact of a knockout on the biomass production rate, directly identifying metabolic essential genes that can be prioritized as potent drug targets to sensitize resistant cells.

### 3.2 Deep learning and foundation models for perturbation prediction

As biology moves beyond linear modeling, the focus has shifted toward data-driven approaches exemplified by generative foundation models. Unlike mechanistic models that require explicit biochemical rules, these architectures ingest massive single-cell multi-omics datasets to construct universal cell embeddings. While early deep learning frameworks provided a starting point, current trends involve models like scGPT and GearNet, which utilize pre-trained cell embeddings to learn the language of gene expression.

A defining advantage of these foundation models is their dual capability for zero-shot and fine-tuned perturbation predictions. In a zero-shot setting, models such as scGPT leverage their vast pre-training on diverse cell atlases to predict the effects of unseen perturbations exemplified by the knockout of genes not explicitly included in the training data by navigating the learned latent space of gene regulation (30). Conversely, fine-tuned prediction allows these architectures to be specialized for specific biological contexts, a process exemplified by Geneformer, which can be refined with context-specific datasets to identify dosage-sensitive genes or predict emergent resistance mechanisms in a particular tumor subtype (31). By refining the pre-trained weights with smaller, high-fidelity datasets, fine-tuning significantly enhances the accuracy of predicting localized signaling responses compared to generic models.

For instance, transformer-based perturbation frameworks have been employed to reveal ligand-centered activation mechanisms in T cells within the colorectal cancer microenvironment (32). Furthermore, deep learning architectures are now being used for large-scale in silico screening of anticancer drugs at the single-cell level, identifying specific vulnerabilities such as the EZH2 or PLK1 pathways (33). By disentangling baseline cellular states from perturbation-induced effects, these AI frameworks capture complex non-linear compensatory mechanisms, predicting how bypass pathways might be activated following a primary gene knockout.

### 3.3 Spatial in silico perturbations and microenvironmental modeling

The integration of spatial transcriptomics has expanded the definition of *in silico* knockout from the intracellular level to the tissue architecture level. This approach allows for the simulation of perturbations within specific anatomical niches, such as the tumor-stroma interface. Rather than just removing a gene, researchers can simulate the removal of specific cell populations or the blockade of cell-cell communication pathways. For example, large-scale single-cell analysis combined with *in silico* perturbation has been used to dissect the dynamic evolution of hepatocellular carcinoma, identifying specific metabolic and inflammatory meta-programs that drive metastasis (16). Similarly, by virtually knocking out the secretion of ligands like Wnt5a from hypoxia-induced inflammatory fibroblasts (18) or targeting the ADAM12-mediated myofibroblast program (34), one can observe the subsequent state changes in adjacent malignant cells. This reveals the niche-dependency of tumor progression, providing a theoretical roadmap for identifying checkpoints that impede anti-tumor immunity.

## 4 Applications of *In silico* knockout in tumor immunology

The integration of *in silico* knockout technology into tumor immunology has revolutionized our ability to dissect the complex interactions within TME. By leveraging computational models to simulate the loss of specific genetic components, researchers can predict immune responses and identify therapeutic vulnerabilities with unprecedented precision.

### 4.1 Identification of key immune regulatory genes

*In silico* perturbation serves as a high-throughput discovery engine for identifying genes that govern the balance between immune activation and suppression. To identify immune suppressor genes, researchers utilize deep learning frameworks like scGPT or SCENIC+ to simulate the deletion of myeloid-specific factors. For instance, simulating the knockout of the E3 ubiquitin ligase Cop1 has been shown to alter the secretion of Ccl2 and Ccl5, thereby reducing the infiltration of pro-tumorigenic macrophages and enhancing anti-tumor immunity (35). This computational prediction was subsequently validated through *in vivo* CRISPR-Cas9 screens and bone marrow chimeras, confirming that Cop1 deficiency significantly inhibits tumor growth by remodeling the myeloid compartment. Conversely, the search for immune activator genes often involves perturbing metabolic or signaling checkpoints. Recent simulations of mannose metabolism pathways revealed that disrupting specific metabolic nodes can reshape T cell differentiation, effectively turning exhausted populations into potent effectors (36). These findings were physically corroborated by dietary mannose supplementation in tumor-bearing mice, which demonstrated a marked increase in CD8+ T cell effector function and improved response to anti-PD-1 therapy.

### 4.2 Analysis of immune evasion mechanisms

Virtual knockout techniques allow for the systematic deconstruction of the pathways tumors use to bypass immune surveillance. In the context of the PD-1/PD-L1 pathway, *in silico* models can predict the downstream transcriptional shifts that occur when these checkpoints are blocked, helping to identify resistance signatures. This approach is particularly effective for studying T cell exhaustion, where simulating the knockout of transcription factors like TOX or specific kinases such as CHEK1 reveals how the epigenetic landscape of a T cell can be reprogrammed from a dysfunctional state back to a functional one (37). Specifically, the predicted role of CHEK1 as an immunosuppressive driver was clinically reinforced by the observation that high CHEK1 expression in lung adenocarcinoma patients correlates with poor prognosis and reduced immune cell infiltration.

### 4.3 Discovery of immunotherapy targets

The transition from discovery to clinical application is accelerated by using virtual perturbations to prioritize checkpoint inhibitors and combination therapies. Beyond classic PD-1/CTLA-4 targets, *in silico* screens have identified stromal checkpoints such as ADAM12 in fibroblasts; simulating its loss predicts a significant increase in CD8+ T cell infiltration into previously cold tumors (34, 38). This stromal dependency was physically validated using patient-derived organoids and mouse models, where the pharmacological inhibition of the ADAM12-mediated myofibroblast program successfully restored T cell access to the tumor core. Furthermore, virtual knockout facilitates the prediction of synergistic effects in combination therapy. By simulating the simultaneous inhibition of a metabolic regulator and a signaling receptor, models can identify which dual-targeting strategies most effectively overcome the immunosuppressive barriers of the TME without the exhaustive cost of physical combinatorial libraries.

### 4.4 Regulation of cell-cell communication at the single-cell level

At the resolution of single-cell transcriptomics, virtual knockout is used to perturb ligand-receptor networks to understand microenvironment remodeling. Tools like CellChat, when combined with perturbation modules, allow researchers to delete a specific ligand (such as Wnt5a or MIF) from a tumor cell and observe the ripple effect across the entire communication interactome (19). For example, the virtual removal of THBS2+ cancer-associated fibroblasts (CAFs) or SPP1+ macrophages has demonstrated how these specific sub-populations act as hubs for immunosuppression. The functional importance of these predictions was substantiated by clinical spatial profiling, which revealed that the physical co-localization of SPP1+ macrophages and ITGA5+ fibroblasts is a deterministic feature of metastasis in hepatocellular carcinoma (39). Their removal from the model results in a complete restructuring of the spatial and chemical signals that normally exclude T cells, providing a theoretical roadmap for precision fibroblast-targeting strategies.

## 5 Discussion

The transition from descriptive omics to predictive *in silico* perturbation represents a paradigm shift in cancer immunotherapy. However, the maturation of this field requires addressing fundamental tensions between computational abstraction and biological complexity.

### 5.1 Schools of thought: mechanistic rigor vs. data-driven scalability

A significant divergence exists in how the field approaches the digital twin of the TME. One school of thought prioritizes mechanistic rigor, utilizing GEMs and FBA to simulate perturbations within biologically defined stoichiometric constraints. Proponents argue that these models provide causal transparency that black-box AI cannot match. Conversely, an emerging school of thought champions data-driven scalability, leveraging transformer-based foundation models like scGPT to learn regulatory grammars directly from massive single-cell repositories. The controversy lies in whether the latent spaces of AI truly capture biological causality or merely sophisticated correlations. Resolving this tension likely requires hybrid architectures where mechanistic laws act as stabilizers for AI-driven predictions.

### 5.2 Current research gaps: beyond transcriptional hegemony

Despite the proliferation of single-cell datasets, a critical research gap persists in the cross-layer fidelity of virtual knockouts. Most current models are anchored in transcriptional abundance, yet mRNA levels often correlate poorly with protein activity or metabolic flux due to post-translational modifications. This domain gap means that a virtual knockout of a transcript might ignore the functional resilience provided by protein stability or metabolic bypasses. To address this deficiency, the integration of multimodal technologies such as Cellular Indexing of Transcriptomes and Epitopes by Sequencing (CITE-seq) into existing frameworks is essential (40). By providing simultaneous quantification of surface protein expression and RNA abundance, CITE-seq data allow virtual knockout models to anchor transcriptional perturbations in concrete protein-level changes, thereby refining the predictive accuracy of signaling state transitions. Furthermore, incorporating emerging single-cell proteomic signatures enables the modeling of post-translational regulatory logic, a process facilitated by frontier architectures like SCN-β that leverage multi-scale embeddings to bridge the gap between gene expression and functional protein activity (41). Future models must transition from purely transcriptomic transformers to multimodal architectures capable of cross-referencing these diverse data streams to ensure that a simulated genetic loss reflects true biochemical ablation. Furthermore, while we can simulate the loss of a single gene, we currently lack the computational capacity to model the combinatorial curse of dimensionality, specifically how the simultaneous perturbation of multiple checkpoints within diverse spatial niches, including the interaction between ADAM12+ fibroblasts and SPP1+ macrophages, reshapes the global immune landscape. Accompanying this biological complexity is a significant escalation in hardware requirements, as the traditional curse of dimensionality has effectively transitioned into a curse of GPU memory. Running large-scale foundation models like Geneformer or scGPT for extensive perturbation screens necessitates substantial computational infrastructure, often requiring high-performance A100 or H100 GPU clusters to handle the quadratic scaling of self-attention mechanisms (31). This creates a practical barrier for many academic laboratories, where the memory intensity of processing millions of cells across thousands of simulated gene knockouts can exceed available local resources. Addressing this requires not only more powerful hardware but also the development of memory-efficient algorithms, such as FlashAttention or quantized low-rank adaptation, to democratize access to frontier in silico knockout frameworks (42).

### 5.3 Bridging the discrepancy: from digital simulations to biological reality

A fundamental debate centers on the equivalence of virtual and physical ablation. Computational models typically simulate a perfect loss of function, whereas real-world CRISPR-Cas9 or pharmacological interventions involve varying degrees of efficiency, off-target effects, and systemic toxicity. Current simulations often operate in a fluid digital environment, largely failing to account for the biophysical constraints of the 3D tumor architecture, such as interstitial pressure and oxygen gradients. Bridging this gap requires the integration of 4D spatiotemporal modeling, where the digital twin evolves not just in its molecular state, but also within the physical and mechanical pressures of the evolving tumor ecosystem. Beyond physical constraints, a critical frontier involves transitioning from static snapshots to models that capture the temporal dynamics of clonal evolution and the emergence of drug resistance. Current efforts are beginning to integrate longitudinal single-cell sequencing data with mathematical modeling of evolutionary fitness, a process exemplified by frameworks like CloneAlign that link chromosomal copy number alterations to transcriptional shifts (43). Future digital twins must incorporate these longitudinal trajectories to simulate how specific perturbations, such as the knockout of a primary oncogenic driver, might inadvertently select for resistant sub-clones or activate latent compensatory pathways over time. By utilizing multi-time-point data to parameterize these models, researchers can move toward 4D simulations that predict not only the immediate response to therapy but also the long-term evolutionary bottlenecks that lead to clinical relapse (44).

### 5.4 Potential future developments: toward autonomous discovery

The future of *in silico* perturbations lies in the move toward autonomous, closed-loop discovery systems. We anticipate the development of self-correcting models that utilize active learning to suggest the most informative wet-lab experiments, which in turn refine the model’s predictive accuracy. Furthermore, the integration of multi-modal foundation models incorporating spatial transcriptomics, digital pathology, and clinical longitudinal data will allow for the simulation of personalized therapeutic journeys. By transitioning from static snapshots to dynamic, high-fidelity simulations that capture non-linear stochasticity and phenotypic plasticity, *in silico* frameworks will eventually move beyond being mere screening tools to becoming the central engine for precision oncology and the design of synergistic combination immunotherapies.
